# Editorial: Biomimetic Materials for Tissue Regenerations

**DOI:** 10.3389/fcell.2022.825455

**Published:** 2022-03-02

**Authors:** Mariappan Rajan, Stevo Najman, Naresh Kumar Rajendran

**Affiliations:** ^1^ Department of Natural Products Chemistry, School of Chemistry, Madurai Kamaraj University, Madurai, India; ^2^ Faculty of Medicine, University of Nis, Rochester, NY, United States; ^3^ School of Medicine, University of Rochester, Rochester, NY, United States

**Keywords:** biomaterails, bone regenaration, osteoblast, cartilage, cell adhesion and migration

In tissue engineering, the ultimate goal is to engineer an entire functioning organ, which requires building complex structures of different tissue types. They resemble the natural formations of organs, cells have to be correctly located relative to each other. It has been shown in cocultures that cells could show spontaneous tissue-like organization when seeded concurrently into the scaffold. The ideal scaffolds should have an interconnected porous structure, well-designed pore size, and adequate porosity not only to allow cell attachment, proliferation, and differentiation, butalso the effective bioactive agents and nutrient exchange during new tissue development. A three-dimensional scaffold is seeded with desired cell types, while an individual organ cell is a specific mechanism for the construction or regeneration of the cells. Particularly, bone organ development at the affected place eventually reduces *in situ* and is repaired with the newly generated bone cells.

Artificial scaffolds have been applied and used as a supporting structure for cell cultures and the domination of cell growth in repairing impaired tissues or organs. During cell regeneration, the scaffold temporarily helps in cell regeneration. It gradually biodegrades either in the healing process or after, producing a new tissue with a desired shape and properties. The challenge of tissue engineering is to mimic what happens in nature. Attempts are being made to engineer *in vitro* practically every tissue and organ in the body. Work is proceeding in creating tissue-engineered liver, nerve, kidney, intestine, pancreas, and even heart muscle and valves. In connective tissues, work has been ongoing worldwide for many years in the engineering of tendon, ligament, bone, and cartilage. Recently, there have many reports of success in skin, bladder, airway, and bone, where tissue-engineered constructs have been used successfully in patients. As the subject of this special issue is the collection of body organ regeneration materials and their Cell Adhesion and Migration for the development and regeneration of the tissues, in this regard biomaterials are significantly involved in the development of the repair of bone tissues. The collected articles report the use and efficacy of different materials like scaffold, composite, 3 days materials to the repair and development of osteoblast, cartilage repair, chondrocytes, and etc. Meanwhile, a review by Tang et al. discusses the recent trends in the development of bone regenerative biomaterials and the use of traditional and modern bone defect repair biomaterials for bone tissue regeneration (Tang et al.).

Zhu et al. presented the potential of biomimetic intrinsically disordered proteins as bone graft materials (Zhu et al.). Two biomimetic peptides (P2 and P6) are incorporated into the SmartBonePepR composite to increase the bioactivity of the bone regeneration ability. The SmartBonePepR composite proved multimodal biological effects as good viability, proliferation rate on human MSC cells *in-vitro* cell analysis, and gene expression analysis. Diwu et al. reported a perfect mimic of human bone material such as selenium substituted Hydroxyapatite (HAP-Se) covered by lactic acid (LA)—Polyethylene glycol (PEG)—Aspartic acid (AS) composite with the loading of vincristine sulfate (VCR) drug (HAP-Se/LA-PEG-AS/VCR) was fabricated for twin purposes of bone regenerations (Wang et al.). The HAP-Se/LA-PEG-AS/VCR composite was coated on a titanium implant through electrophoretic deposition (EPD). The porous nature of the composite has expressed the more exceptional biocompatibility in bone cells and toxicity with the cancer cells of prepared composites. The outcome of the investigation proposed the biomaterial suitable for implantation and helps accelerate bone regeneration on osteoporosis and osteosarcoma affected hard tissue.

Cartilaginous defect repair is difficult because of the avascular nature and limited regeneration of the ability of cartilage *in situ* (Diwu et al.). Autogenous cartilage transplantation, allogenic cartilage transplantation, and artificial substitutes are therapeutic options. An acellular matrix (AM) as a natural biomaterial is gaining increasing attention in tissue engineering applications. An acellular cartilaginous matrix (ACM) and acellular dermal matrix (ADM) are two kinds of the most widely used AMs in cartilage tissue engineering. However, there is still debate over which of these AMs achieves optimal cartilage regeneration, especially in large immunocompetent animals. Wang et al. fabricated porous ADM and ACM scaffolds by a freeze-drying method and confirmed that ADM had a larger pore size than ACM (Ci et al.). By recolonizing with goat auricular chondrocytes and *in vitro* culture, ADM scaffolds exhibited a higher cell adhesion rate, more homogeneous chondrocyte distribution, and neocartilage formation than ACM. Additionally, quantitative polymerase chain reaction (qPCR) indicated that expression of cartilage-related genes, including ACAN, COLIIA1, and SOX9, was significantly higher in the ADM group than the ACM group. In summary, the ADM is appropriate for cartilage regeneration, which can be used for cartilage regeneration in large immunocompetent animals.

Ci et al. *reported* cartilage sheet was prepared into engineered Cartilage gel (ECG) and combined with DBM to explore the feasibility of regenerating 3D cartilage with controlled shape and mechanical strength (She et al.). The authors introduced the new concept for cartilage regenerations by analogous steel reinforced composite packed ECG and DBM materials. A Biomimetic Biphasic Scaffold Consisting of Decellularized Cartilage and Decalcified Bone Matrixes for Osteochondral Defect Repair was reported by Cao et al. and Bosch-Rué et al. This study developed a biomimsubchondraletic biphasic scaffold for OCD repair via an iterative layered lyophilization technique that controlled the composition, substrate stiffness, and pore size in each phase of the scaffold. The biphasic scaffold consisted of a superficial decellularized cartilage matrix (DCM) and underlying decalcified bone matrix (DBM) materials used for osteochondral tissue regenerations. The results demonstrate that the biomimetic biphasic scaffold has a good osteochondral repair effect.

The recent advances in bone regenerations by 3D printed biomimetic scaffold materials are a rapid development of tissue engineering technology, and have provided new methods for tracheal replacement (Cao et al.). A biomimetic scaffold with a separated-ring structure—a polycaprolactone (PCL) scaffold with a ring-hollow alternating structure—was three-dimensionally printed as a framework. The collagen sponge was embedded in the hollows amid the PCL rings by pouring, followed by lyophilization. The experimental results showed substantial deposition of tracheal cartilage and formation of a biomimetic trachea mimicking the native trachea both structurally and mechanically. The investigation highlights the advantage of a biomimetic trachea with a separated-ring structure that mimics the native trachea both structurally and mechanically and demonstrates its promise in repairing long-segment tracheal defects.

